# Long-Term Stability of Blood Serum Biomarkers in Traumatic Brain Injury: A Feasibility Study

**DOI:** 10.3389/fneur.2022.877050

**Published:** 2022-05-18

**Authors:** Harm Jan van der Horn, Koen Visser, Johan Bijzet, Pieter Vos, Joukje van der Naalt, Bram Jacobs

**Affiliations:** ^1^Department of Neurology, University of Groningen, University Medical Center Groningen, Groningen, Netherlands; ^2^Department of Rheumatology and Clinical Immunology, University of Groningen, University Medical Center Groningen, Groningen, Netherlands; ^3^Department of Neurology, Slingeland Hospital, Doetinchem, Netherlands

**Keywords:** biomarkers, inflammation, stability, traumatic brain injury, laboratory, concussion

## Abstract

Few studies on traumatic brain injury (TBI) have investigated the stability of blood serum biomarkers after long-term storage at low temperatures. In the current feasibility study we analyzed acute phase serum samples from patients with mild TBI as well as patients with moderate and severe TBI that were collected more than 10 years ago (*old samples*). We were particularly interested in mild TBI, because injury effects are more subtle in this category as compared to moderate-severe TBI. Therefore, the primary objective was to find out whether several biomarkers were still detectable for these patients. Additionally, we examined whether biomarker levels varied as a function of injury severity. For comparison, we also analyzed samples from an ongoing mTBI cohort (*new samples*) and healthy controls. Samples were treated with care and were not being subjected to freeze-thaw cycles. We measured concentrations of interleukins (IL6 and 10) and brain specific markers (total tau, UCH-L1, GFAP, and NF-L). No significant differences in biomarker concentrations were found between old and new mild TBI samples. For IL6, IL10, and UCH-L1 higher concentrations were found in moderate and severe TBI as compared to mild TBI. In conclusion, our study shows that long-term storage does not rule out the detection of meaningful biomarker concentrations in patients with TBI, although further research by other laboratories is warranted.

## Introduction

The continuing mission of many research teams around the world is to find biomarkers for traumatic brain injury (TBI). Several biomarkers are already in use (S-100B in Scandinavia) or approved (glial fibrillary acidic protein (GFAP) and ubiquitin carboxyl-terminal hydrolase (UCH)-L1 in the U.S.) for CT-scan triaging at emergency departments (1, 2). Research has also shown promising results for various brain-specific and inflammatory biomarkers regarding the prediction of outcome after TBI (3, 4). Despite these considerable achievements, little is known about the stability of biomarkers after long-term storage. This is a very relevant topic because serum/plasma samples are usually aliquoted into smaller volumes of which some might be stored in freezers for many years. Furthermore, research is increasingly done in multicenter context where a substantial amount of data is collected and biomaterials may be stored for a long time before analyses are conducted (5, 6).

It goes without saying that leftover samples are ideally used to answer future research questions. Also from an ethical perspective is it imperative to make full use of all biomaterials that patients were willing to donate. Concerns about whether or not long-term storage of samples will significantly affect the integrity of biomarkers, and may render them undetectable, are still not fully settled. Recent research in TBI has shown that over the course of 3 days, concentration of GFAP, UCH-L1 and S100B remain stable when stored at 4–5°C (7). However, whether storage for many years will affect biomarker concentrations still remains a question.

In the current short communication we report the results of a feasibility study set out to assess the influence of long-term storage of serum samples on the stability of a small set of promising brain-specific and inflammatory biomarkers in patients with TBI. We were especially interested in mild TBI, since injury effects in these patients are less clear as compared to moderate and severe TBI. We analyzed samples from patients with mild TBI as well as patients with moderate and severe TBI, that were collected over 10 years ago, to find out whether biomarkers were still detectable, and if concentrations varied as a function of injury severity. We compared biomarker concentrations in old mild TBI samples with those in newer samples obtained from patients included in an ongoing prospective study on mild TBI.

## Methods

### Participants

Venous blood samples were obtained in the first 12 h post-injury from: (1) 80 patients with mild (*n* = 12), moderate (*n* = 26), and severe (*n* = 42) TBI included in the Radboud University Brain Injury Cohort Study (RUBICS) study in the period 2006–2009 (“old” samples); (2) 49 patients with mild TBI (mTBI) and 10 healthy controls (HC) included in the AIM-TBI study (Dutch trial registry no. NL8484), in the period 2020–2021 (samples we will refer to as “new” samples). Criteria for a diagnosis of mild, moderate or severe TBI in the RUBICS study were previously described by Jacobs et al. (8). A diagnosis of mTBI in the AIM-TBI study was made according to the criteria of the American Congress of Rehabilitation Medicine (9). Ethical approval for blood collection in the RUBICS study was given by the ethical committee of the Radboud University Medical Center (Radboudumc), The Netherlands (AMO 04/064 and CMO 2004/025); approval for the AIM-TBI study was given by the medical ethical committee of the University Medical Center Groningen (UMCG), The Netherlands (METc 2018/681). All patients or next of kin provided written informed consent. All procedures were carried out in accordance with the 1964 Helsinki Declaration.

### Sample Processing, Storage, and Analyses

Old samples were allowed to clot for 30 min after collection, centrifuged at 1,000 g and serum was then stored at −40°C at the Radboudumc until 2014. Subsequently, they were transferred to the UMCG and stored at −80°C until current analyses. Samples were never thawed before analyses. New samples were allowed to clot for 60 min after collection, and then centrifuged at 1,400 g. Subsequently, serum was aliquoted, and stored at −80°C. Interleukin (IL)-6 (lower limit of detection (LLD) 1.4 pg/mL, limit of quantification (LOQ) 1.5 pg/mL, inter-variation coefficient (IVC) 2.9%), IL-10 (LLD 1.2 pg/mL, LOQ 1.3 pg/mL, IVC 4.6%), total tau (LLD 10 pg/mL, LOQ 11 pg/mL, IVC 1.8%), and UCH-L1 (LLD 305 pg/mL, LOQ 307 pg/mL, IVC 2.3%) concentrations were determined (single measurement on 2 days) using a Luminex Human Discovery multiplex assay (R&D Systems, Oxford, UK) on May 18, 2021, and June 6, 2021, in accordance with the manufacturer's instructions. Levels of GFAP (LLD 20 pg/mL, LOQ 20 pg/mL, IVC 3.4%) and neurofilament light (LLD 7.8 pg/mL, LOQ 20 pg/mL, IVC 4.6%) were determined in duplo using the Human GFAP DuoSet ELISA (R&D Systems, Oxford, UK) and Cusabio Human Neurofilament protein L ELISA kit (Bio-Connect Services, Huissen, The Netherlands), respectively, both according to manufacturer's instructions (all GFAP measurements were done on 1 day, NFL was measured on 2 days). The selection of cytokines was based on a recent systematic review on blood-based inflammatory biomarkers in mTBI that was published by our research group, showing the potential value of IL6 and IL10 (10); selection of brain-specific markers was based on studies that were published in recent years (11, 12). The lab technician who measured biomarker levels was blinded for clinical parameters.

### Statistical Analyses

Statistical analyses were performed using the Statistics and Machine Learning Toolbox implemented in MATLAB v2020a (Natick, MA, USA). For nominal variables Chi square tests were conducted. As all continuous variables were non-normally distributed, Kruskal-Wallis tests were used to test for group differences (HC vs. mTBI-new, vs. mTBI-old, vs. modTBI-old, vs. sevTBI-old), which in case of significance was followed by *post-hoc* group comparisons (MATLAB *multcompare* function). In addition, biomarker concentrations were compared between male and female subject for every subgroup. Spearman correlations were computed between biomarker concentrations and age for every subgroup. Alpha was set at 0.05. *Post-hoc* group comparisons were corrected for multiple comparisons using Tukey's test. Data plots were made using *notBoxPlot* (v1.31) implemented in MATLAB.

## Results

### Participant Characteristics

Groups were roughly matched for age and sex (Table 1). As expected, the moderate and severe TBI groups more frequently had intracranial traumatic lesions, as well as more severe polytrauma [expressed by the Injury Severity Score (ISS)] than the mild patients.

**Table 1:** Demographics, clinical parameters, and biomarker results.

	**“Old”**			**“New”**			
	**Mild**	**Moderate**	**Severe**	**Mild**	**HC**	***Test statistic***	***p***
	**(*n* = 12)**	**(*n* = 26)**	**(*n* = 42)**	**(*n* = 49)**	**(*n* = 10)**		
Age, y, mdn (range)	55 (22–71)	35 (18–70)	34 (18–66)	33 (18–72)	32 (24–44)	H = 8.92	0.082
Sex, % female	33	58	29	39	60	χ^2^ = 6.59	0.11
GCS-score, mdn (range)	15 (13–15)	11 (9–12)	3 (3–8)	15 (14–15)	N/A	H = 113.83	<0.001
LOC, % yes	50	77	100	74	N/A	χ^2^ = 15.95	<0.001
PTA, % yes	75	100	100	90	N/A	χ^2^ = 7.64	0.004
Lesions acute CT, % yes	17	73	86	18	N/A	χ^2^ = 52.73	<0.001
Injury mechanism					N/A	χ^2^ = 17.05	0.148
Falls, %	58	23	19	25			
Traffic, %	25	65	67	65			
Sports, %	17	8	7	4			
Assault, %			5				
Other, %		4	2	6			
Interval injury to blood sampling, min., mdn (range)	160 (60–675)	75 (30–205)	80 (10–599)	147 (23–361)	N/A	H = 15.46	0.001
ISS, mdn (range)	16 (4–33)	21 (4–50)	35 (17–59)	7 (4–21)	N/A	H = 80.2	<0.001
**Biomarker concentrations, Mdn, (range)**							
IL-6	23.54 (1.38–467.1)	24.64 (1.38–3803.9)	41.56 (1.55–903.3)	5.33 (1.38–42.9)	1.38 (1.38–2.2)	H = 56.18	<0.001
IL-10	1.4 (1.2–48.9)	1.97 (1.2–436.8)	4.03 (1.2–255.6)	1.2 (1.2–36.5)	1.2 (1.2–3.0)	H = 17.55	0.002
Total tau	10 (10–10)	10 (10–25.1)	10 (10–10)	10 (10–30.4)	10 (10–10)	H = 2.09	0.72
UCH-L1	305 (305–9,020)	332.89 (305–26,192)	1186.57 (305–25,181)	305 (305–341)	305 (305–305)	H = 53.69	<0.001
GFAP	20 (20–924)	20 (20–649)	20 (20–800)	20 (20–779)	20 (20–840)	H = 1.8	0.77
NF-L	2,786 (2,176–4,633)	2,958 (1,303–5,545)	2,638 (1,267–5,672)	2,573 (856–5,545)	2,631 (1,004–4,330)	H = 6.11	0.19

*CT, computed tomography; GCS, Glasgow Coma Scale, GFAP, glial fibrillary acidic protein; IL, interleukin; ISS, Injury Severity Score, LOC, loss of consciousness; Mdn, median; min, minutes; NF-L, neurofilament light; PTA, post-traumatic amnesia; UCH, ubiquitin carboxyl-terminal hydrolase; y, years*.

### Biomarker Levels

**Figure 1:**
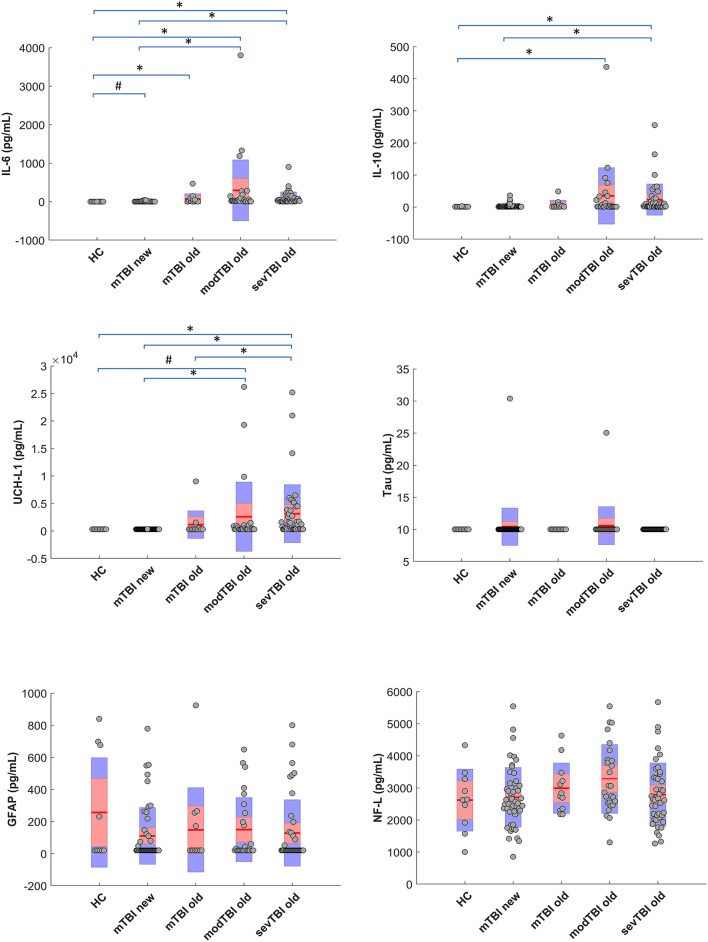
Serum biomarker concentrations.

Significant group differences for UCH-L1 (*p* < 0.0001), IL-6 (*p* < 0.0001), and IL-10 (*p* = 0.001) were found. Figure 1 shows the data for the different groups as well as the *post-hoc* group differences. It should be noticed that no differences were found between the old and new mTBI groups for none of the markers. No significant group differences were found for GFAP, tau, or NFL. The association between biomarker levels and interval *injury-blood sampling* is depicted in Supplementary Figure 1. When correcting for multiple comparisons, no significant correlations between biomarkers levels and age were found for any of the subgroups; also no significant differences were found between male and female subjects.

## Discussion

To the best of our knowledge, this is the first study in TBI research to report biomarker data from serum samples that have been stored for over 10 years. Based on our results, we have reason to believe that biomarker concentrations can still be reliably measured after long-term storage. In particular, we have found elevations of interleukins and UCH-L1 concentrations that varied as a function of TBI injury severity.

Stability of biomarkers in TBI and other neurologic disorders is still a subject of discussion. Although the stability in TBI has been examined in the post-acute phase, data on long-term preservation in still lacking (7). Research findings in non-TBI populations suggest that NFL, GFAP, and total tau are stable for years when stored at −80°C (7, 13). Interestingly, we still found increased values for IL-6 and IL-10 in old samples of patients with TBI, which is in contrast to results from studies in non-TBI samples that have demonstrated that cytokines are only stable up to 2 years when stored at −80°C (14). The absence of elevations for NFL, GFAP, and total tau as compared to healthy controls in our study is an interesting finding, and could have been caused by long-term storage, but also by the choice of assay used. Many different assays are being used to determine biomarkers in TBI, each with its own detection and quantification limits, which may lead to the differences in literature. For instance, the LLD for NFL (7.8 pg/mL), GFAP (20 pg/mL), total tau (10 pg/mL), and UCH-L1 (305 pg/mL) for the assays used in our study is relatively high compared to some of the assays used by other studies (0.29, 8, 0.02, and 45 pg/mL, respectively) (2, 3, 12), which might have contributed to the absence of a significant elevation in mild TBI. Therefore, further studies on long-term biomarker stability in TBI are warranted that use assays with lower LLD. On a side note, it has also been shown that GFAP is detectable in a subgroup of the healthy population, which could be an explanation for the elevations in 4 of our 10 healthy controls (15–17).

Biomarker kinetics, and thus the interval between injury and blood sampling, also determines biomarker concentrations (12, 18). For example, research by Papa and colleagues has demonstrated that UCH-L1 peaks early after injury, while GFAP starts to rise at ~4 h after injury in mild/ moderate TBI (12). Furthermore, a recent study on the temporal profile of biomarkers in sports-related concussion suggests that elevations of GFAP, NFL and tau are more likely to occur even days after injury, although there was not an acute measurement in that study (11). This might explain our null findings for GFAP, NFL and tau, as samples were collected relatively early after injury: For the mild TBI patients (for both the old and new cohorts), the majority of samples was collected under 4 h post injury (median of 160 and 147 min, respectively). For the moderate and severe patients, samples were obtained even earlier, with majority of blood samples drawn under 2 h. It is also important to realize that biomarker levels at a certain time point after TBI reflect both the circulating proteins, due to the initial injury, as well as proteins that are being released due to ongoing (secondary) pathological process (19). Furthermore, degradation processes may vary due to non-injury related factors, such as glomerular filtration rate.

There are several limitations of our study that need to be mentioned. First, there were no earlier analyses done on the “old” samples when they were still relatively new, so a direct comparison of biomarker levels cannot be made. Second, there were no healthy controls included in the “old” cohort. Third, the healthy control and “old” mild TBI groups in our study were relatively small, and groups were not optimally matched (with respect to age, sex). Fourth, the older samples were stored at −40°C for several years, which theoretically might have led to a reduced stability of biomarkers. Lastly, although our study was not designed to thoroughly examine pre-analytical factors, we acknowledge the large variability in biomarker concentrations due to differences in other pre-analytical factors such as blood collection method, preparation of serum, time allowed for clotting to take place, tube type, storage temperature, transportation, and various more (19, 20).

In aggregate, the current feasibility study shows that long-term storage does not preclude the measurement of meaningful biomarker concentrations in patients with TBI, provided that samples are treated with care and are not being subjected to freeze-thaw cycles, although more research is needed, from other laboratories, to confirm our results. Future studies also need to take into account the various (pre-) analytical variables that are known to affect measured biomarker concentrations.

## Data Availability Statement

Data supporting the conclusions of this article will be made available by the authors upon reasonable request.

## Ethics Statement

The studies involving human participants were reviewed and approved by Radboud University Medical Center (Radboudumc), Netherlands. The patients/participants provided their written informed consent to participate in this study.

## Author Contributions

HH, BJ, and JN designed and planned the experiments. KV and JB conducted the laboratory experiments. HH conducted statistical analyses and wrote the initial draft. All authors read and critically revised the manuscript.

## Conflict of Interest

The authors declare that the research was conducted in the absence of any commercial or financial relationships that could be construed as a potential conflict of interest.

## Publisher's Note

All claims expressed in this article are solely those of the authors and do not necessarily represent those of their affiliated organizations, or those of the publisher, the editors and the reviewers. Any product that may be evaluated in this article, or claim that may be made by its manufacturer, is not guaranteed or endorsed by the publisher.
