# Supplementary figures and images for: Long-Term Stability of Blood Serum Biomarkers in Traumatic Brain Injury: A Feasibility Study
Source: Front Neurol. 2022 May 18;13:877050. doi: 10.3389/fneur.2022.877050 (PMC9158477; doi:10.3389/fneur.2022.877050)

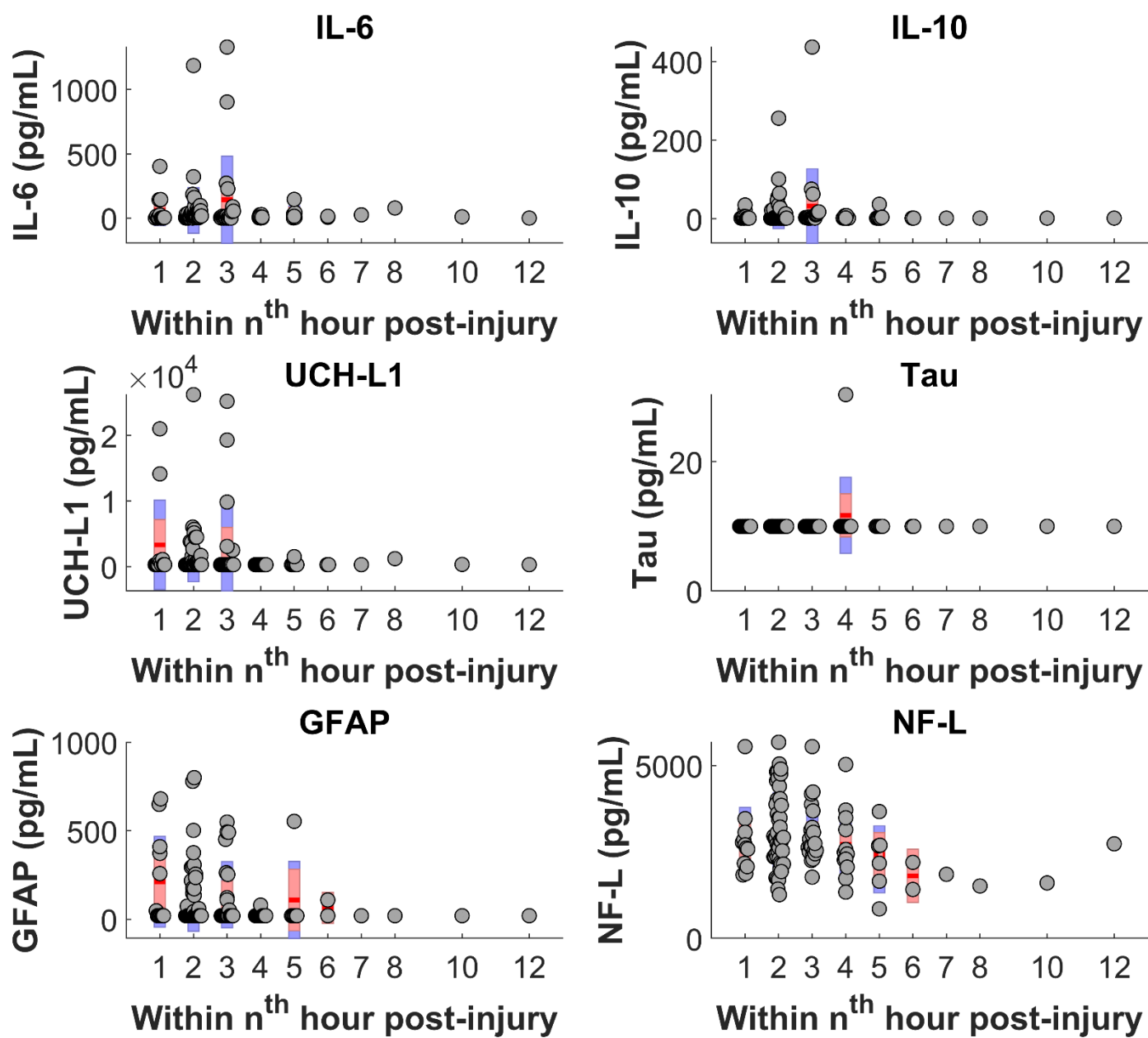

**Supplementary Figure 1.** Biomarkers levels related to interval injury-to-blood-sampling.

Supplement: Supplementary file 1 [file Data_Sheet_1.PDF]
